# Progress Toward Eradication of Polio — Worldwide, January 2011–March 2013

**Published:** 2013-05-03

**Authors:** Polio Eradication Dept, World Health Organization, Geneva, Switzerland, Div of Viral Diseases, National Center for Immunization and Respiratory Diseases, Global Immunization Div, Center for Global Health, CDC

In May 2012, the World Health Assembly of the World Health Organization (WHO) declared the completion of polio eradication a programmatic emergency (1). Since the launch of the Global Polio Eradication Initiative (GPEI) in 1988, the number of annual polio cases has decreased by >99%. As of March 2013, circulation of indigenous wild poliovirus (WPV) continued in only three countries: Afghanistan, Nigeria, and Pakistan (the last case in India had onset in January 2011). This report provides an update on progress toward global polio eradication during January 2011–March 2013, using data reported as of April 23, 2013 (2). The number of WPV cases reported globally decreased 66%, from 650 in 2011 to 223 in 2012; WPV cases decreased 53% (from 80 to 37) in Afghanistan and 71% (from 198 to 58) in Pakistan, but increased 97% (from 62 to 122) in Nigeria. The number of imported WPV cases in previously polio-free countries decreased from 309 in 12 countries in 2011 to six in two countries in 2012 (3,4). During January–March 2013, a total of 22 WPV cases were reported worldwide, compared with 48 cases during the same period in 2012. An estimated 2.05 billion doses of oral poliovirus vaccine (OPV) were administered in 2012 to approximately 448 million persons, primarily children aged <5 years, in supplemental immunization activities (SIAs) conducted in 46 countries. SIAs were temporarily suspended in areas of Pakistan and Nigeria after attacks against health workers occurred in December 2012 and the first quarter of 2013. The number of confirmed WPV cases has decreased to the lowest level ever, but security concerns continue to threaten the overall goal of global eradication.

## Routine Vaccination Coverage

By the end of 2011, the latest complete year for which data are available, infant routine vaccination coverage worldwide with 3 doses of poliovirus vaccine by age 12 months (Pol3) was estimated at 84%. By WHO region, coverage with Pol3 was 76% in the African Region, 93% in the Region of the Americas, 83% in the Eastern Mediterranean Region, 94% in the European Region, 74% in the South-East Asia Region, and 96% in the Western Pacific Region (4). Coverage varied substantially among and within countries. Estimated national Pol3 coverage was 66% in Afghanistan, 75% in Pakistan, and 73% in Nigeria 2011 (4), with substantial variability within each country.

## Extent of SIAs

In 2012, SIAs using OPV were conducted in 46 countries against WPV and circulating vaccine-derived polioviruses (cVDPV) (6). The SIAs included 77 national immunization days, 120 subnational immunization days, 29 child health days, and nine mop-up rounds. A total of 67 SIAs were conducted in Afghanistan, India, Nigeria, and Pakistan, 46 SIAs in previously polio-free countries affected by outbreaks or reestablished transmission following importations, and 122 preventive SIAs in countries with no WPV cases during 2012. An estimated 2.05 billion doses of OPV were administered to approximately 448 million persons, primarily children aged <5 years. Of these doses, 869 million were trivalent OPV, 1.1 billion were bivalent OPV, and 36 million were type 1 monovalent OPV. Short-interval additional dose SIAs, implemented in rapid succession (<2 weeks apart) to quickly raise immunity using monovalent OPV and/or bivalent OPV, became a core strategy in Pakistan in 2011 in high-risk areas and continued during 2012; these measures were introduced in Afghanistan during 2013 in low-performing districts. SIAs were temporary suspended in some areas of Pakistan and Nigeria during December 2012 and the first quarter of 2013 following attacks against health workers; SIAs were resumed after security precautions were enhanced for vaccination teams.

## Poliovirus Surveillance

WPV transmission is monitored through surveillance for acute flaccid paralysis (AFP) cases and testing of stool specimens in WHO-accredited laboratories. AFP surveillance performance is monitored by using standard indicators for sensitivity and timeliness (nonpolio AFP rate and stool specimen adequacy). In 19 countries with transmission of poliovirus (WPV and/or cVDPV) during 2011–2012, national AFP surveillance performance indicators were met in 12 (63%) countries in 2011 and 13 (68%) countries in 2012. Indicators improved from 2011 in several high-risk countries in close proximity to Nigeria (Angola, Central African Republic, and Democratic Republic of the Congo [DRC]), but not in others (Chad and Niger) (7). AFP cases caused by cVDPV were detected in eight countries in 2012 (Afghanistan, Chad, DRC, Kenya, Nigeria, Pakistan, Somalia, and Yemen) (7).

## Reported WPVs

Of 223 cases reported in 2012, a total of 202 were WPV type 1 (WPV1), and 21 were WPV type 3 (WPV3), decreases of 65% and 69%, respectively, compared with 2011. During January–March 2013, a total of 22 WPV1 cases were reported globally from three countries, representing a 54% decrease compared with the 48 WPV1 cases reported during the same period in 2012 from four countries (Table). As of February 2012, India no longer was considered to be polio-endemic. During January–March 2013, fewer WPV cases were reported in Afghanistan, Nigeria, and Pakistan (two, 14, and six, respectively) than during the corresponding period in 2012 (six, 24, and 15, respectively). As of April 23, no WPV3 cases had been reported globally in 2013.

What is already known on this topic?

Since the launch of the Global Polio Eradication Initiative in 1988, the number of polio cases have decreased by >99%, and more than 100 countries have stopped transmission. However, circulation of wild poliovirus (WPV) has continued uninterrupted in three countries: Afghanistan, Nigeria, and Pakistan. In previous years, WPV has spread from polio-endemic countries to neighboring countries and sometimes beyond. Twelve previously polio-free countries had WPV circulation in 2011.

What is added by this report?

The number of polio cases confirmed globally and the geographic extent of WPV transmission has reached the lowest levels ever reported. In 2012, only Afghanistan, Chad, Niger, Nigeria, and Pakistan reported polio cases. Except for Nigeria, where cases nearly doubled compared with 2011, the number of cases in each country decreased. During January–March 2013, the number of polio cases in Afghanistan, Nigeria, and Pakistan were lower than during the same period in 2012. However, security risks following attacks on health workers delivering polio vaccine have impeded progress in certain areas of Pakistan and Nigeria.

What are the implications for public health practice?

In areas of Pakistan and Nigeria, special security measures have been undertaken to sustain progress toward polio eradication such as the protection of vaccinators by law enforcement officers. Increasing local community engagement in security-compromised areas is critical to overcoming inaccessibility and insecurity and enhancing community vaccine acceptance. Efforts are under way to further focus resources on high-risk areas to interrupt transmission.

## Polio-Endemic Countries

### Afghanistan

In 2012, a total of 37 WPV1 cases were reported, a 54% decrease from 80 cases reported in 2011. No WPV3 cases have been reported from Afghanistan since April 2010.

### Nigeria

In 2012, a total of 122 WPV cases (103 WPV1 and 19 WPV3) were reported, a 97% increase from 62 cases (47 WPV1 and 15 WPV3) reported in 2011. The most recently reported WPV3 case from northern Nigeria occurred in November 2012.

### Pakistan

In 2012, a total of 58 WPV cases (55 WPV1, two WPV3, and one WPV1/WPV3 mixed infection) were reported compared with 198 cases (196 WPV1 and two WPV3) in 2011, a 71% decrease. No WPV3 cases have been reported since April 2012 in the Federally Administered Tribal Areas of Pakistan.

## Polio-Nonendemic Countries

The number of WPV cases resulting from importations and outbreaks in previously polio-free countries decreased from 309 in 12 countries* in 2011 to six in two countries in 2012 (Niger and Chad) (Figure). In Niger, one WPV1 case was reported in 2012, compared with five WPV1 cases reported during 2011. All of the virus isolates from persons with WPV1 in Niger during 2011–2012 were genetically related to WPV1 circulating in Nigeria. In Chad, which experienced reestablished transmission after WPV1 importation in 2010 (3), five WPV1 cases were reported in 2012, compared with 132 WPV1 cases reported in 2011, a 96% decrease. WPV1 was detected in sewage samples through environmental surveillance in Cairo, Egypt, during December 2012 and was linked genetically to WPV1 circulating in Sindh, Pakistan, during 2012; WPV has not been detected in Egypt in environmental samples or AFP cases since December 2012. No new WPV outbreaks have been reported in polio-free countries globally in 2013, as of April 23.

### Editorial Note

After the May 2012 World Health Assembly resolution, the implementation of the GPEI Global Emergency Action Plan 2012–2013 and national emergency action plans in countries with WPV transmission led to substantial progress toward global polio eradication. Since the resolution, the number of WPV cases reported globally and the geographic extent of WPV transmission have reached the lowest levels ever reported. The possible interruption of WPV3 transmission in Asia and the prevention and control of WPV outbreaks in previously polio-free countries are important achievements. Sustained efforts are needed in polio-free countries at risk for outbreaks after WPV importation, including maintaining population immunity, and conducting vigilant surveillance.

Key elements of national emergency plans have included enhanced government commitment to polio eradication, increased vaccination coverage through routine and supplementary immunization efforts (e.g., improved micro-planning, effective strategies to vaccinate children previously missed, and enhanced monitoring of SIA quality), increased accountability at all administrative levels, improved partner coordination (e.g., polio operations rooms at national and state levels), and the implementation of innovative approaches (e.g., short-interval additional dose SIAs). Other critical program efforts include increases in technical support and human resources provided to priority countries through the placement of thousands of additional polio staff members at the lowest administrative levels. Further technical support was provided by an expansion and increase in duration of the international Stop Transmission of Polio (STOP) program,† in both polio-endemic and polio-nonendemic, high-risk countries, and national STOP (N-STOP) programs, in Nigeria and Pakistan, that develop sustained national capacity and expertise (8).

Security remains a problem in the polio-endemic areas of Afghanistan and in areas of Pakistan. New security risks following attacks on health workers delivering polio vaccine have impeded progress in certain areas of Pakistan and Nigeria. In these locations, national governments have implemented special security measures, such as the protection of vaccinators by law enforcement officers. Increasing local community engagement with field staff members in security-compromised areas is critical to overcoming inaccessibility and insecurity and increasing community vaccine acceptance. Strategies also have been implemented to identify and vaccinate chronically missed children, reduce parental refusals, maintain sufficient vaccine supplies, and focus resources in countries and regions at the greatest risk for outbreaks (9).

At the request of the World Health Assembly, GPEI has developed a Polio Eradication and Endgame Strategic Plan (2013–2018), in consultation with stakeholders, to complete polio eradication and transition GPEI infrastructure (10). Main objectives of the plan include 1) detecting and interrupting WPV and cVDPV poliovirus transmission by strengthening global surveillance, enhancing SIA quality, and preventing and rapidly responding to outbreaks; 2) strengthening immunization systems and withdrawing OPV by increasing routine vaccination coverage, ensuring the availability and use of appropriate polio vaccines; 3) ensuring laboratory containment of poliovirus and certifying WPV eradication; and 4) transitioning GPEI assets and infrastructure within routine immunization programs and leveraging programmatic lessons. As highlighted by the cessation of WPV transmission in India, commitment and dedication to program implementation have achieved successes; however, the challenges that remain to complete global polio eradication require sustained commitment and continued coordinated efforts.

## Figures and Tables

**FIGURE:**
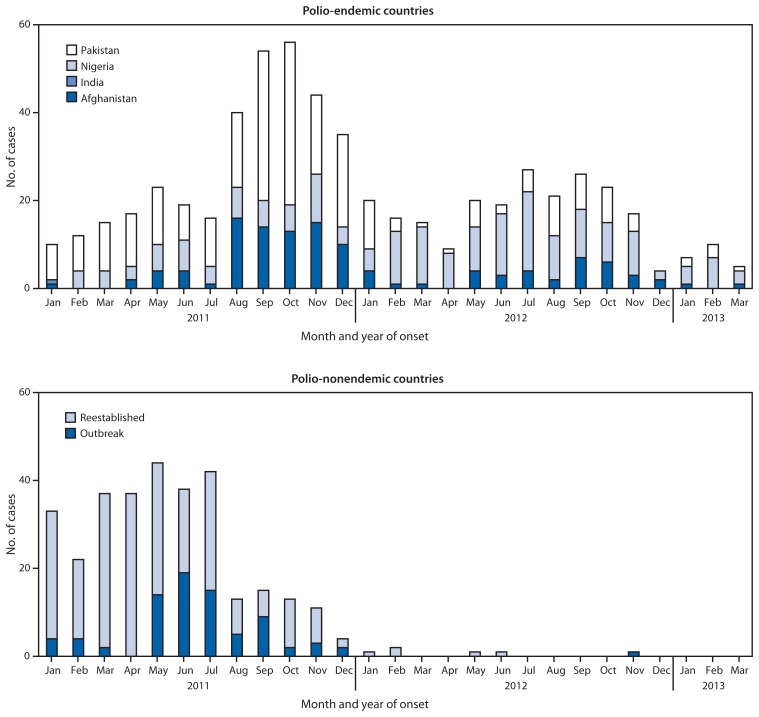
Number of reported cases of wild poliovirus infection among polio-endemic countries and polio-nonendemic countries, by month and year of onset — January 2011–March 2013^*^ ^*^ Data as of April 23, 2013.

**TABLE:** Number of reported cases of wild poliovirus (WPV) infection, by country and serotype — January 2011–March 2013*

Country	2011	2012	2012 Jan–Mar	2013 Jan–Mar
**Polio-endemic**				
Afghanistan	80	37	6	2
India	1	0	0	0
Nigeria	62	122	24	14
Pakistan	198	58	15	6
**Polio-nonendemic**				
Angola	5	0	0	0
Central African Republic	4	0	0	0
Chad	132	5	3	0
China	21	0	0	0
Côte d’Ivoire	36	0	0	0
DRC	93	0	0	0
Gabon	1	0	0	0
Guinea	21	0	0	0
Kenya	1	0	0	0
Mali	7	0	0	0
Niger	5	1	0	0
Republic of Congo	1	0	0	0
**Total**	**650**	**223**	**48**	**22**
**Total WPV type 3**	**67**	**21**	**8**	**0**
**Total WPV type 1**	**583**	**202** †	**40** †	**22**

**Abbeviation:** DRC = Democratic Republic of the Congo.

* Data as of April 23, 2013.

† Includes one case mixed infection types 1 and 3 WPV.

## Footnotes

* Angola, Central African Republic, Chad, China, Côte d’Ivoire, Democratic Republic of the Congo, Gabon, Guinea, Kenya, Mali, Niger, and Republic of the Congo.

† Additional information available at http://www.cdc.gov/polio/stop.
